# Preclinical imaging of kallikrein-related peptidase 2 (hK2) in prostate cancer with a ^111^In-radiolabelled monoclonal antibody, 11B6

**DOI:** 10.1186/s13550-014-0051-5

**Published:** 2014-09-19

**Authors:** Oskar Vilhelmsson Timmermand, David Ulmert, Susan Evans-Axelsson, Kim Pettersson, Anders Bjartell, Hans Lilja, Sven-Erik Strand, Thuy A Tran

**Affiliations:** Department of Medical Radiation Physics, Lund University, Barngatan 2:1, S-211 85 Lund, Sweden; Department of Surgery (Urology), Memorial Sloan-Kettering Cancer Center, 1275 York Avenue, New York, NY 10065 USA; Department of Clinical Sciences, Div. Urological Cancers, Lund University, Jan Waldenstroms gata 5, S-205 02 Malmö, Sweden; Department of Biochemistry and Food Chemistry/Biotechnology, University of Turku, Tykistökatu 6A BioCity, 205 20 Turku 52 Turku, Finland; Department of Laboratory Medicine and Medicine (GU Oncology), Memorial Sloan-Kettering Cancer Center, 1250 York Avenue, New York, NY 10065 USA; Nuffield Department of Surgical Sciences, University of Oxford, Oxford, UK; Department of Laboratory Medicine, University Hospital UMAS, Lund University, Malmö, Sweden; Lund University Bioimaging Center, BMC, Lund University, Klinikgatan 32, S-222 42 Lund, Sweden

**Keywords:** Prostate cancer, Human kallikrein-related peptidase 2, Human kallikrein gene family, ^111^In-DTPA-11B6, Molecular imaging

## Abstract

**Background:**

Prostate cancer is a leading cause of death in the male population of the western world. Human kallikrein-related peptidase 2 (hK2) is abundantly expressed in malignant prostatic tissue, and its gene, *KLK2*, is regulated by the androgen receptor. 11B6 is a murine IgG_1_ monoclonal antibody directed against free human hK2. In this study, we performed a preclinical evaluation of ^111^In-labelled 11B6 in mouse xenografts to investigate its potential in the clinical staging and assessment of metastatic prostate cancer.

**Methods:**

11B6 was radiolabelled with ^111^In through CHX-A″-DTPA chelation. *In vivo* biodistribution and uptake of ^111^In-DTPA-11B6 were measured until 168 h post-injection in NMRI nude mice bearing subcutaneous LNCaP xenografts. The binding specificity to hK2 was evaluated by both *in vivo* competitive binding assays with excess non-labelled 11B6 and hK2-negative DU145 xenografts. SPECT/CT imaging of subcutaneous and intra-tibial LNCaP xenografts was used to visualize the tumours.

**Results:**

Tumour uptake of ^111^In-DTPA-11B6 in LNCaP xenografts was 19% ± 0.78%IA/g at 48 h, giving a tumour-to-blood ratio of 1.6, which increases to 2.4 at 1 week post-injection. Accumulation was low in other organs except for the salivary glands, which is probably the result of cross-reactivity with mouse kallikreins. Significantly lower tumour accumulation was observed in competitive assays and DU145 xenografts. SPECT/CT imaging could clearly visualize the subcutaneous and intra-tibial LNCaP xenografts.

**Conclusions:**

Our study demonstrates the potential of ^111^In-DTPA-11B6 for the detection of metastatic prostate cancer and monitoring anti-androgen therapy, as it exhibits an increased uptake and accumulation in viable tumour when compared to normal tissue. A humanised version of the 11B6 monoclonal antibody is currently under evaluation.

**Electronic supplementary material:**

The online version of this article (doi:10.1186/s13550-014-0051-5) contains supplementary material, which is available to authorized users.

## Background

Although improvements have been made in approaches for both diagnosis and treatment, prostate cancer (PCa) remains the second leading cause of cancer-related deaths in the male population of the western world [1]. The ratio of local-regional PCa to that of metastatic disease has increased since the introduction of prostate-specific antigen (PSA) testing, subsequently raising questions regarding overdiagnosis [2,3]. Nevertheless, there is a need for new molecular imaging moieties to improve the clinical staging and monitoring of PCa in order to identify the optimal course of treatment for the disease.

Today, molecular imaging methods based on metabolic markers such as ^18^F-FDG (fluorodeoxyglucose) and ^18^F/^11^C-Choline are commonly used; however, there are known limitations for these tracers. ^18^F-FDG works well in a more advanced disseminated disease, but is ineffective in detecting localized PCa because of its lower glycolytic uptake compared to other neoplasms [4]. Also, the normal elimination of ^18^F-FDG through the urinary bladder can mask the uptake in the prostate and regional lymph nodes [5]. ^18^F/^11^C-Choline is the most common radiotracer in PCa imaging today, and while they perform better than ^18^F-FDG, they are still limited in their ability to differentiate between a localized PCa and benign disease [6].

Molecular imaging of androgen receptor (AR) signalling holds the promise for more accurate disease evaluation and therapeutic monitoring, with additional implications for hormonal treatment and radiation therapy in PCa. PCa growth is dependent on androgens that signal through the AR and plays a fundamental role in cancer cell proliferation, apoptosis and invasion/metastasis [7]. A newly developed radiotracer, ^18^F-FDHT (16β-[^18^F]fluoro-5α-dihydrotestosterone), enables the imaging of AR expression. Furthermore, preliminary clinical studies demonstrated that ^18^F-FDHT might provide for the imaging of AR expression during disease progression in castration-resistant PCa [8].

The commercially available ProstaScint®, ^111^In-capromab pendetide, targets an intracellular epitope of the prostate-specific membrane antigen (PSMA) [9], another surrogate of AR signalling. Promising results were recently reported using a new antibody (J591) against an extracellular domain of the PSMA molecule and presents as a possibility to suppress PSMA expression following anti-androgen treatments [10]. However, the PSMA expression in other tissues such as the human brain [11] hampers this approach to PCa imaging.

An alternative strategy could be to target AR-dependent antigens such as PSA and hK2 as they express almost exclusively in the prostate tissue [12,13]. Both PSA and hK2 are serine proteases, encoded by the human kallikrein genes *KLK3* and *KLK2* located on chromosome 19, respectively, and are well characterized as AR-regulated genes [14-16]. These kallikreins are produced by the same secretory luminal cells in the prostate and share an 80% amino acid homology, as well as several structural similarities [15-17]. Importantly, the PSA and hK2 antigens are abundantly expressed in malignant prostate tissue throughout all clinical stages. Recent publications on the 5A10 [18] and PSA30 antibodies [19] have explored the concept of PSA imaging, including targeting the free, unbound forms of prostate-specific antigen (fPSA). Results from animal studies using the PSA30 antibody showed a selective uptake in LNCaP tumours *in vivo*; however, the retention was faint and most likely due to shortcomings in the ^125^I-labelling method. In addition, ^89^Zr-labelled 5A10 exhibited better imaging capabilities but was accompanied by increased liver uptake. Nonetheless, this PET tracer was sufficient in detecting AR-dependent changes in PSA expression levels in mouse tumour lesions, as well as in distinguishing PCa cells within bone lesions - both of which may be useful in the staging and clinical evaluation of advanced prostate cancer [18].

Regardless of the similarities between PSA and hK2, hK2 displays properties distinct from those of PSA. hK2 has been used in immunoassays to improve the accuracy of PCa screening [17,20]. In serum, most hK2 is in its free form, although the total levels of hK2 are in the range of 1% total PSA but similar to that of fPSA [21]. As a tissue marker for PCa, the hK2-specific immunostaining pattern differs from that of PSA, with an increased intensity in the PCa tumour and lymph node metastases compared to that observed in benign tissue [22,23]. Moreover, the very-low-to-no expression in other organs [12,13] makes hK2 a potential target candidate for PCa imaging.

Owing to these potentially advantageous characteristics of hK2 as a PCa biomarker, we investigated the possibility of targeting free hK2 in an androgen-dependent PCa model using ^111^In-radiolabelled 11B6 - a murine IgG_1_ hK2-specific monoclonal antibody previously used in immunoassays for free hK2 [24]. Here, we discuss the results of *in vivo* hK2 imaging with ^111^In-labelled 11B6 in AR-positive LNCaP xenografts.

## Methods

### Antibody conjugation and radiolabelling

The murine monoclonal antibody, 11B6, was first described and characterized by Vaisanen et al. [24] and was provided by the University of Turku (Turku, Finland) for this study. Conjugation and radiolabelling was performed as previously described by Tolmachev et al. [25]. Briefly, 2 mg of 11B6 was conjugated with the chelator CHX-A″-DTPA (B-355, Macrocyclics; Dallas, TX, USA) through the isothiocyanate functional group. A solution of 11B6 (4 to 5 mg/mL in PBS) was adjusted to pH 9.2 using 0.07 M sodium borate buffer (Sigma Aldrich; St. Louis, MO, USA). CHX-A″-DTPA was then added to the protein solution at a molar ratio of 3:1 (chelator to antibody) and incubated at 40°C with gentle shaking. The reaction was terminated after 4 h, and CHX-A″-DTPA-11B6, henceforth referred to as DTPA-11B6, was separated from the free chelate by size-exclusion chromatography on a NAP-5 column (GE Healthcare; Uppsala, Sweden) equilibrated with 20 mL of 0.2 M ammonium acetate buffer (Sigma Aldrich), pH 5.5. Conjugated 11B6 was eluted with 1 mL of ammonium acetate buffer, and aliquoted samples were stored at −20°C.

For radiolabelling, approximately 125 μL of DTPA-11B6 (approximately 1 μg/μL in 0.2 M ammonium acetate buffer pH 5.5) was mixed with a predetermined amount (approximately 50 to 100 MBq) of ^111^InCl_3_ (Mallinckrodt Medical; Dublin, Ireland), incubated at room temperature for 1.5 to 2 h and then purified on a NAP-5 column (GE Healthcare) equilibrated with PBS (Thermo Scientific; Waltham, MA, USA). Labelling efficiency and kinetics were monitored by instant thin-layer chromatography (ITLC) (Biodex, Shirley, NY, USA) eluted with 0.2 M citric acid (Sigma Aldrich). In this system, the radiolabelled conjugate remains at the origin line, while free ^111^In and ^111^In-DTPA migrate with the solvent front. The radioactive distribution was determined using a Cyclone Storage Phosphor System with Optiquant quantification software (Perkin Elmer; Waltham, MA, USA).

### Binding kinetics with surface plasmon resonance

The 11B6 binding kinetics were analysed by surface plasmon resonance using a Biacore 2000 (Biacore AB; Uppsala, Sweden). The affinity of 11B6 to hK2 before and after CHX-A″-DTPA conjugation was determined. The hK2 antigen, provided by the University of Turku (Department of Biotechnology; Turku, Finland), was produced and purified as previously described [26]. hK2 antigen (25.9 μg/mL in 10 mM sodium acetate buffer pH 4.0 (Sigma Aldrich)) was immobilized on a CM4 research grade chip (Biacore AB) by amino coupling using N-hydroxysuccinimide (NHS), 1-ethyl-3-(3-dimethylaminopropyl) carbodiimide hydrochloride (EDC) and 1 M ethanolamine hydrochloride-NaOH, pH 8.5, in a Biacore 2000 system. Samples were flown over two flow cells, one being a blank reference, in five different concentrations ranging from 0.5 to 100 nM to detect eventual binding. One of the two flow cells contained immobilized hK2, while the other was served as a blank reference. The binding kinetics were studied in a 3-min-long association phase and a 15-min-long dissociation phase with a flow rate of 30 μL/min, followed by regeneration with 25 mM glycine buffer pH 2.7. Kinetic constants were calculated using a 1:1 Langmuir binding model with correction for mass transfer. BIAEvaluation 4.1 software (Biacore AB) was used for calculations.

### Stability studies

The stability of ^111^In-DTPA-11B6 was assessed in triplicate by incubating the compound at 4°C in PBS buffer or at 37°C in murine serum collected from normal NMRI mice. For stability in PBS, 1 μL (*n* = 3) was taken at 1, 2, 3 and 7 days and analysed by ITLC. For stability in serum, 10 μL of ^111^In-DTPA-11B6 (corresponding to 3 μg of antibody with 0.8 to 0.9 MBq ^111^In) was mixed with 100 μL of mouse serum. Approximately 20 μL of each mixture was collected after 2, 3 and 9 days of incubation and analysed by SDS-PAGE on a NuPAGE 4% to 12% Bis-Tris gel (Invitrogen; Carlsbad, CA, USA) in MES buffer (200 V constant, approximately 30 min). ^111^In-DTPA and free ^111^In diluted in PBS were run in parallel with the incubated sample as controls. The distribution of the samples along the gel was evaluated using a Cyclone Storage Phosphor System (Perkin Elmer).

### Cell lines

LNCaP and DU145 were purchased from American Type Culture Collection (ATCC; Manassas, VA, USA) and cultured in RPMI 1640 medium (Thermo Scientific) supplemented with 10% foetal bovine serum (Thermo Scientific) with 100 U/mL penicillin and 100 μg/mL streptomycin (Thermo Scientific). The cells were maintained at 37°C in a humidified incubator at 5% CO_2_ and were detached with trypsin-EDTA solution (Thermo Scientific).

### Animal models

All animal experiments were conducted in compliance with the national legislation on laboratory animals' protection and with the approval of the Ethics Committee for Animal Research (Lund University, Sweden). Two animal models were used in this study, NMRI-Nu with subcutaneous (s.c.) xenografts and SCID mice with intra-tibial xenografts. NMRI-Nu mice (6-to-8-week-old, Taconic; Ry, Denmark) were inoculated in the right flank by s.c. injection of 5 to 8 × 10^6^ cells in a 200 μL of cell suspension of 1:1 mixture of medium with Matrigel (BD Biosciences; San Jose, CA, USA). Tumours were allowed to develop for 6 to 8 weeks. SCID mice (6-to-8-week-old male, Charles River; Charles River, NJ, USA) were maintained under isoflurane anaesthesia during surgery. For intra-tibial inoculations, the tibia was punctured using a 23-gauge needle, and 1 × 10^5^ LNCaP cells were injected into the tibial cavity. The puncture was closed with bone wax, the incision sutured and the animals received a palliative dose of Temgesic (Buprenorphine, RB Pharmaceuticals; Richmond, VA, USA) once daily for 3 days post-surgery. Intra-tibial tumours were allowed to develop for 8 to 10 weeks. Additionally, a group of normal NMRI mice (*n* = 4) were used to study the distribution of the tracer in healthy animals. Animals were euthanized by intraperitoneal (i.p.) injection with 20 μL per gram of body weight Ketalar-Rompun solution. (Ketalar, 10 mg/mL; Pfizer; New York, NY, USA, and Rompun, 1 mg/mL; Bayer Animal Health; Monheim, Germany).

### Biodistribution studies

Biodistribution studies were conducted to evaluate the uptake of ^111^In-DTPA-11B6 in human prostate cancer LNCaP xenografts. Mice (*n* = 3 to 5 per time point) received ^111^In-DTPA-11B6 (0.4 to 0.6 MBq, 20 μg of mAb, in approximately 100 μL of PBS) through intravenous (i.v.) tail vein injection. Blood and organs (including tumour) were taken at 4, 24, 48, 72 and 168 h post-injection, weighed and measured in a NaI(TI) well counter (Wallac Wizard 1480 Wizard, Perkin Elmer). The activity injected into each animal was measured and used to determine the count rate, in comparison with a standard solution of ^111^In-DTPA-11B6. Data were corrected for background and physical decay.

Organ-specific uptake values were calculated as percent injected activity per gram of tissue (%IA/g) or percent injected activity (%IA). Among the organs resected were the lateral and ventral prostate, from now on referred to as prostate, and the submandibular glands, from now on called salivary glands.

### *In vivo* binding specificity

*In vivo* competitive binding studies were performed to investigate the specificity of ^111^In-DTPA-11B6 to hK2. A 40-fold excess of non-labelled 11B6 was i.v. injected as a co-injection or at 168, 120 and 48 h prior to an i.v. injection of ^111^In-DTPA-11B6 in hK2-positive LNCaP xenografts (*n* = 3 to 4 per pre-injection time point). Blood and organs (including tumour) were taken at 48 h post-injection of ^111^In-DTPA-11B6, weighed and analysed as above. The binding specificity was also evaluated by measuring the uptake of ^111^In-DTPA-11B6 in hK2-negative DU145 xenografts expressing low levels of hK2 (*n* = 3) at 48 h post-injection, which are considered to be hK2-negative when compared to other PCa tumours.

### Small animal PET/SPECT/CT/MR imaging

Animals were anaesthetized with 2% to 3% isoflurane gas (Baxter; Deerfield, IL, USA) for all imaging purposes. For SPECT/CT imaging, NMRI-nu mice with s.c. LNCaP xenografts (48 h post-injection, *n* = 4; 72 h post-injection, *n* = 3; pre-dosed 11B6, *n* = 4; co-injection 5A10, *n* = 3) and SCID mice (*n* = 3) with intra-tibial LNCaP xenografts were i.v. injected with approximately 8 MBq of ^111^In-DTPA-11B6 (approximately 20 μg of mAb in 150 μL of PBS) and imaged, for 1 h, by using a preclinical SPECT/CT scanner (NanoSPECT/CT Plus, Bioscan; Washington, DC, USA) with the NSP-106 multi-pinhole mouse collimator. SPECT data were reconstructed using HiSPECT software (SciVis; Goettingen, Germany). CT imaging was done before each whole-body SPECT.

Pre-dosed mice were given 0.8 mg of non-labelled mAb 48 h prior to injection of radiolabelled mAb. Co-injections with ^111^In-DTPA-11B6 and 1.5 mg of fPSA-specific 5A10 were done to evaluate the possible cross-reactivity of ^111^In-DTPA-11B6 with PSA.

The legs of the SCID mice were resected after imaging, and the radioactivity in the intra-tibial xenografted and the non-xenografted leg were measured in the NaI(Ti) well counter. Radiolabelling for SPECT of intra-tibial xenografts demonstrated 95% radiochemical purity and was injected directly without NAP-5 column purification. Verification of the intra-tibial tumour growth was performed by MR imaging. The legs were imaged in an 11.7 T (500 MHz for protons) vertical bore MR camera (Agilent Technologies; Palo Alto, CA, USA) equipped with Varian 88/55 micro-imaging triple axis gradient coil (1 T/m maximum gradient strength). Samples were placed in the centre of a Millipede imaging probe (Agilent Technologies; Santa Clara, CA, USA), with an inner diameter of 40 mm.

For PET/CT imaging, mice with LNCaP xenografts were i.v. injected with approximately 12 MBq ^18^F-FDG (*n* = 4) or approximately 12 MBq^18^F-Choline (*n* = 4) and imaged 1 h post-injection using a Bioscan NanoPET/CT Plus preclinical scanner for approximately 15 min. Both SPECT/CT and PET/CT images were analysed using InVivoScope 2.0 software (inviCRO; Boston, MA, USA), and ROIs were drawn using the CT image as anatomical reference.

### Autoradiography and staining

After SPECT imaging at 48 and 72 h, s.c. tumours were resected and embedded in Tissue-Tek® O.C.T™ compound (Sakura Finetek; Alphen aan den Rijn, The Netherlands) and frozen on dry ice. The frozen samples were cryosectioned with a thickness of 20 μm for autoradiography analysis on a Cyclone Storage Phosphor System. The tumour sections were stained with Mayer's hematoxylin and chromotrope 2R, Ch2R (both from Histolab; Gothenburg, Sweden), and scanned using a light-microscope slide scanner (Mirax Midi, Carl Zeiss; Oberkochen, Germany). Thresholds for the autoradiograms were set in ImageJ v.1.47.

### Statistical analysis

Data was analysed using the unpaired, two-tailed Student's *t* test (Microsoft Excel or GraphPad Prism v.4). Differences at the 95% confidence level (*P* < 0.05) were considered to be statistically significant. Figures were produced with GraphPad Prism v.4 (GraphPad Software). All biodistribution data are shown as an average %IA/g of 3 to 5 animals ± SD (standard deviation) unless otherwise stated.

## Results

### Radiolabelling and stability

A radiolabelling yield of 58.6% ± 12.5% (*n* = 3) was achieved. The radiochemical purity after NAP-5 purification was >99% and for labelling with higher activities for imaging a specific activity of 0.30 ± 0.03 MBq/μg (*n* = 3). ^111^In-DTPA-11B6 was stable in PBS buffer at 4°C and in mouse serum at 37°C. More than 95% of the radioactivity was still attached to the radioconjugate after 1 week in both conditions.

### Binding kinetics with surface plasmon resonance

The binding kinetics of 11B6 were analysed on a Biacore instrument. The affinity of 11B6 was high for hK2 and, although conjugation seemed to affect the association constant (k_a_) and the dissociation constant (k_d_), the calculated binding affinities (K_D_; calculated as k_d_/k_a_) were similar. The K_D_ for 11B6 and DTPA-11B6 to hK2 were estimated to be (5.1 ± 2.2) × 10^−11^ M and 6.6 × 10^−11^ ± 0.46 × 10^−11^ M, respectively. Representative sensorgrams of the 11B6 and DTPA-11B6 binding kinetics are shown in the supplementary data (Additional file 1: Figure S1A,B).

### Biodistribution studies

**Figure 1:**
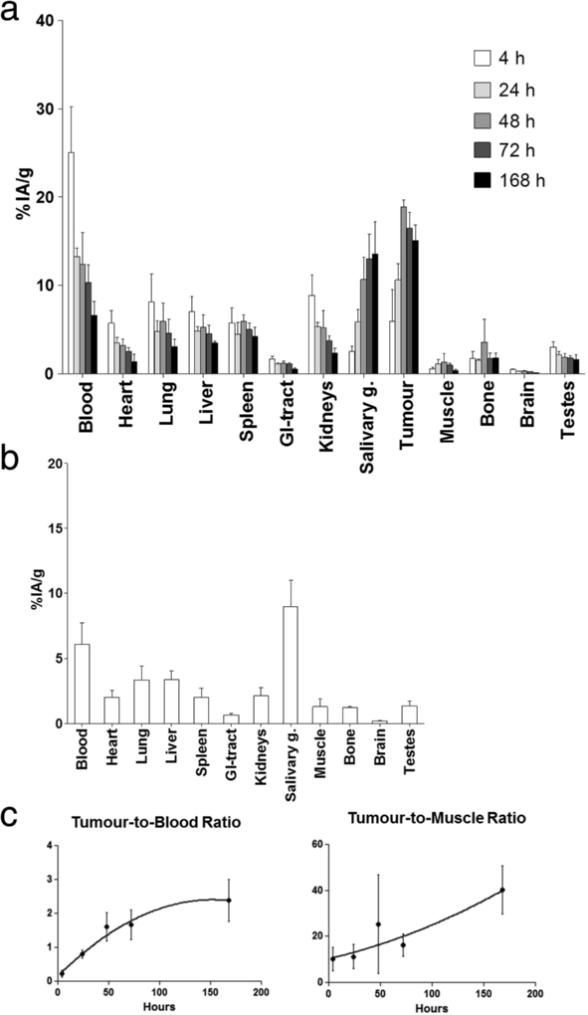
**Biokinetics of** ^**111**^ **In-DTPA-11B6. (a)** Biodistribution of ^111^In-DTPA-11B6 in NRMI nude mice carrying LNCaP xenografts. %IA/g ± SD, 3 to 5 animals per time point. **(b)** Biodistribution of ^111^In-DTPA-11B6 in normal NMRI mice at 48 h. An overall lower %IA/g was observed as compared to tumour-bearing mice but with a retained high uptake in the salivary glands. **(c)** Left, tumour-to-blood ratio is steadily increasing over the time of the experiment. Right, tumour-to-muscle ratio. This high, increasing ratio explains the good visualization and contrast observed in SPECT/CT images. (See Figure 3)

**Figure 2:**
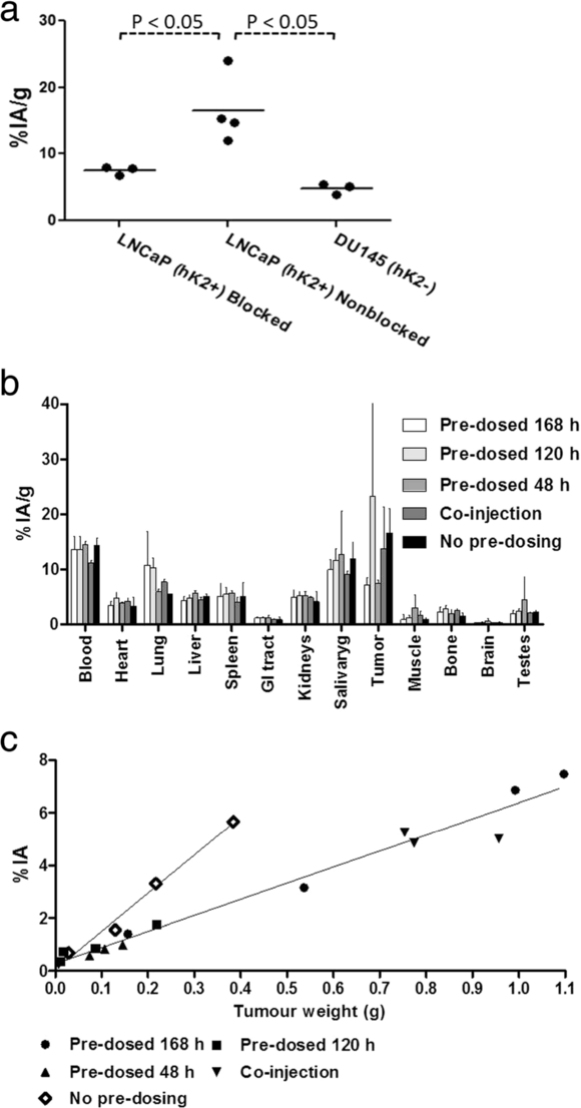
***In vivo*** **binding specificity of** ^**111**^ **In-DTPA-11B6 in NRMI nude mice. (a)** Dot plot of tumour accumulation in blocked LNCaP, non-blocked LNCaP xenografts (hK2+) and DU145 (hK2−). There was a significant difference in active uptake between non-blocked LNCaP xenografts and the other groups. **(b)** Distribution of ^111^In-DTPA-11B6 48 h post-injection in LNCaP xenografted NMRI mice, shown as %IA/g ± SEM with pre-dosing of 0.8 mg of cold 11B6 antibody. **(c)** Dot plot with linear regression for pre-dosing and no pre-dosing as function of tumour weight. This shows that %IA increases with tumour weight for both pre-dosed and normal uptake but that the uptake increases more in tumours with no pre-dosing. The slope calculated from linear regression was 6.1 ± 0.27%IA/g for the pre-dosed groups and 14 ± 1.2%IA/g with no pre-dosing. The R^2^ value was 0.97 for the pre-dosed groups and 0.99 for xenografts without pre-dosing. As %IA/g increases with smaller tumour volume this could explain the large difference seen in (b) between the different pre-dosing groups. By analysing %IA instead, it seems that the effect of pre-dosing is retained over the studied time interval and that 11B6 in fact has a long tumour retention.

The biodistribution of ^111^In-DTPA-11B6 in NMRI mice with LNCaP xenografts revealed that the accumulation in tumour tissue increased threefold from 4 h (5.9 ± 3.6%IA/g) to a maximum at 48 h (19 ± 0.78%IA/g) (Figure 1a). Blood radioactivity was steadily cleared from the circulation over time, resulting in a tumour-to-blood ratio of 1.6 after 3 days and 2.4 at 1 week (Table 1). All organs showed constant, low uptake and low tissue-to-blood ratios (Ti/B), except for the salivary glands and highly vascularized organs, such as the spleen, liver and lung, suggesting no active antibody accumulation. Furthermore, the spleen, liver and lung showed steady increase in tumour-to-tissue (T/Ti) ratios. The T/Ti ratios increased more than threefold between 4 h and 1 week (Table 2). The salivary gland accumulation followed a different pattern, more similar to that of the tumour, reaching a maximum at 1 week (13.6 ± 3.6%IA/g). This elevated uptake could be due to cross-reactivity of ^111^In-DTPA-11B6 since most mice kallikreins are abundant in this organ [27], although mice are naturally deficient hK2 [16]. The high level of mice kallikreins in the salivary glands could be an explanation to retained salivary gland uptake even after pre-dosing (Figure 2b). A similar, but slightly lower, salivary gland uptake (9.0 ± 2.0%IA/g) was seen in tumour-free NMRI mice at 48 h (Figure 1b). These mice exhibited a lower overall organ uptake of the tracer, which could be explained by the lack of shedded antigen circulating in the blood.

**Table 1:** **Tissue-to-blood (Ti/B) ratios of** ^**111**^ **In-DTPA-11B6 in NMRI nude mice carrying LNCaP xenografts**

	**4 h**	**24 h**	**48 h**	**72 h**	**168 h**
Tumour	0.23 ± 0.10	0.80 ± 0.12	1.6 ± 0.41	1.7 ± 0.44	2.4 ± 0.62
Heart	0.23 ± 0.02	0.26 ± 0.03	0.26 ± 0.03	0.24 ± 0.02	0.22 ± 0.16
Lung	0.33 ± 0.12	0.36 ± 0.07	0.48 ± 0.12	0.43 ± 0.07	0.47 ± 0.05
Liver	0.28 ± 0.04	0.36 ± 0.02	0.43 ± 0.03	0.44 ± 0.04	0.55 ± 0.14
Spleen	0.23 ± 0.06	0.34 ± 0.09	0.50 ± 0.09	0.49 ± 0.07	0.64 ± 0.06
GI tract	0.07 ± 0.004	0.08 ± 0.003	0.09 ± 0.01	0.11 ± 0.04	0.08 ± 0.01
Kidneys	0.35 ± 0.05	0.40 ± 0.03	0.41 ± 0.05	0.37 ± 0.04	0.35 ± 0.03
Salivary	0.10 ± 0.01	0.44 ± 0.08	0.86 ± 0.11	1.3 ± 0.11	2.1 ± 0.38
Muscle	0.02 ± 0.003	0.08 ± 0.03	0.09 ± 0.05	0.09 ± 0.02	0.06 ± 0.01
Bone	0.07 ± 0.03	0.12 ± 0.01	0.34 ± 0.34	0.17 ± 0.05	0.27 ± 0.07
Brain	0.02 ± 0.003	0.02 ± 0.003	0.02 ± 0.003	0.02 ± 0.001	0.02 ± 0.003
Prostate	0.06 ± 0.01	0.24 ± 0.04	0.14 ± 0.04	0.19 ± 0.04	0.20 ± 0.05
Testes	0.12 ± 0.02	0.16 ± 0.02	0.15 ± 0.02	0.17 ± 0.02	0.27 ± 0.20

**Table 2:** **Tumour-to-tissue (T/Ti) ratios of** ^**111**^ **In-DTPA-11B6 in NMRI nude mice carrying LNCaP xenografts**

	**4 h**	**24 h**	**48 h**	**72 h**	**168 h**
Heart	0.99 ± 0.44	3.0 ± 0.13	6.2 ± 1.5	6.7 ± 1.7	38 ± 58
Lung	0.75 ± 0.43	2.3 ± 0.21	3.5 ± 1.4	3.7 ± 1.4	5.2 ± 1.5
Liver	0.79 ± 0.34	2.2 ± 0.44	3.8 ± 0.96	3.5 ± 1.0	4.4 ± 0.28
Spleen	0.99 ± 0.45	2.5 ± 0.46	3.2 ± 0.27	3.1 ± 0.57	3.7 ± 0.83
GI tract	3.5 ± 1.6	9.8 ± 1.6	17 ± 3.5	14 ± 1.3	30 ± 8.2
Kidneys	0.62 ± 0.25	2.0 ± 0.24	3.9 ± 1.2	4.2 ± 0.71	6.8 ± 1.5
Salivary	2.3 ± 0.98	1.9 ± 0.24	1.8 ± 0.35	1.2 ± 0.32	1.2 ± 0.37
Muscle	10 ± 5	11 ± 5.3	25 ± 21	16 ± 4.8	40 ± 11
Bone	3.5 ± 0.81	7.0 ± 1.4	7 ± 3.9	10 ± 3.6	9.2 ± 2.6
Brain	12 ± 6.6	39 ± 4.5	75 ± 31	74 ± 22	118 ± 13
Prostate	3.7 ± 1.3	3.5 ± 0.99	12 ± 7.0	8.2 ± 3.0	12 ± 1.5
Testes	1.9 ± 0.82	5.0 ± 0.77	11 ± 2.2	9.5 ± 2.4	11 ± 3.9

At 1 week, the steadily increasing T/Ti ratios for blood and muscle were 2.4 ± 0.62 and 40 ± 11, respectively (Table 1, Table 2 and Figure 1c). Particularly high increases in T/Ti were seen for the brain with a T/Ti of 118 ± 13 at the end of the study. The high brain T/Ti could probably be explained by the tight junctions of the epithelial cells of the brain vasculature and blood-brain barrier and then only reflecting the blood content.

### *In vivo* binding specificity

Pre-injection with an excess of cold antibody (0.8 mg of cold mAb) significantly decreased tumour accumulation of the labelled conjugate. The tumour accumulation was 7.2 ± 0.53%IA/g at 48 h post-injection in mice that received a blocking dose of 11B6 and was significantly lower to the 17 ± 5.2%IA/g observed in non-blocked mice (*P* = 0.03) (Figure 2a). Furthermore, the DU145 hK2-negative xenografts showed significantly lower (*P* = 0.01) tumour accumulation for ^111^In-DTPA-11B6, 4.8 ± 0.86%IA/g compared to 17 ± 5.2%IA/g at 48 h (Figure 2a).

The difference in specific uptake between tumour samples from pre-dosed mice (*n* = 16) and the reference group (*n* = 4) deteriorated with decreasing tumour weight, which skewed the tumour uptake data for the 120 h and co-injection time points (Figure 2b). For all groups, the specific uptake increased with tumour volume, but it was less in the pre-dosed groups (Figure 2c). This demonstrates that it is possible to block the uptake with cold 11B6 and remains effective over the span of 1 week. In other organs, no or small deviations from the non-blocked group were observed.

### Small animal PET/SPECT/CT/MR imaging

**Figure 3:**
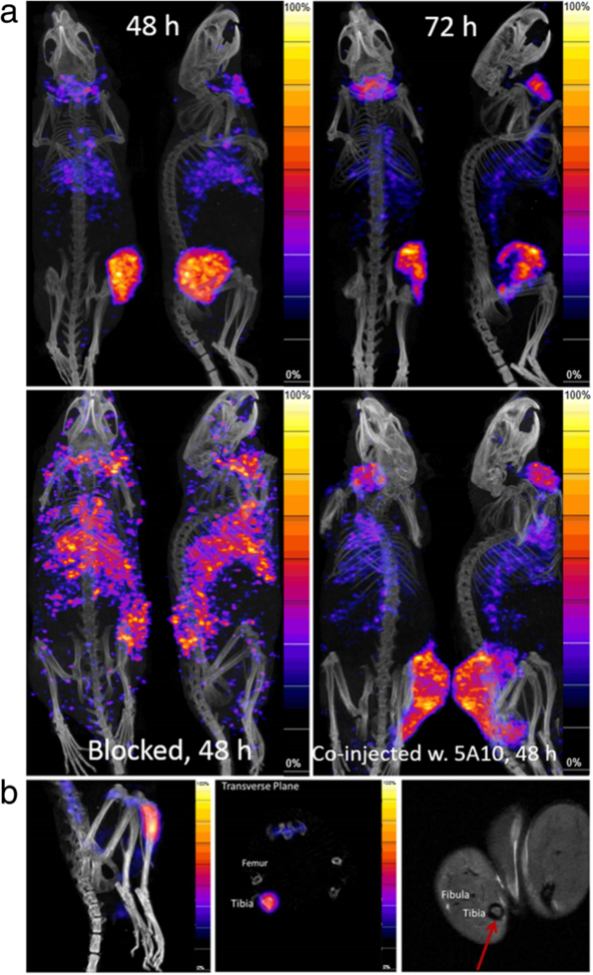
**Preclinical SPECT/CT and MR images. (a)** SPECT/CT images of four NMRI nude mice with LNCaP xenografts on the right flank. Top left, 48 h post-injection; Top right, 72 h post-injection; Bottom left, blocked mouse pre-injected with 0.8 mg of cold antibody 48 h prior to injection of radiolabelled antibody. Bottom right, image of a mouse at 48 h co-injected with 1.5 mg of unlabelled 5A10, which did not block the tumour uptake of ^111^In-DTPA-11B6. It should be noted that for the calculation of the T/B ratio (1.6) in the biodistribution and for the ROI analysis of the SPECT images with a derived T/heart ratio of 1.7, the whole tumour is taken into account. Since the colour scale in the SPECT image is scaled partially based upon high uptake areas of the tumour, a direct comparison between these (which are strikingly visible) and the T/B or T/heart (ROI) is not totally fair. **(b)** SPECT/MR images of SCID mice with intra-tibial xenograft in the left hind limb. Left, SPECT/CT side view, 48 h post-injection; middle, transverse SPECT/CT image slices of the same mouse. The accumulation of ^111^In-DTPA-11B6 in intra-tibial tumours is clearly shown in both images. Right, MR image of the same animal, where the growth of the intra-tibial tumour is well visualized (arrow).

Representative SPECT/CT images of LNCaP xenografts measured 2 to 3 days post-injection of ^111^In-DTPA-11B6 are shown in Figure 3a. The activity distribution in the SPECT images, with a high tumour-to-background contrast, is comparable to the high tumour-to-tissue ratios at these time points (Table 1). The high contrast was most likely attributed to the high affinity and specificity of the tracer.

**Figure 4:**
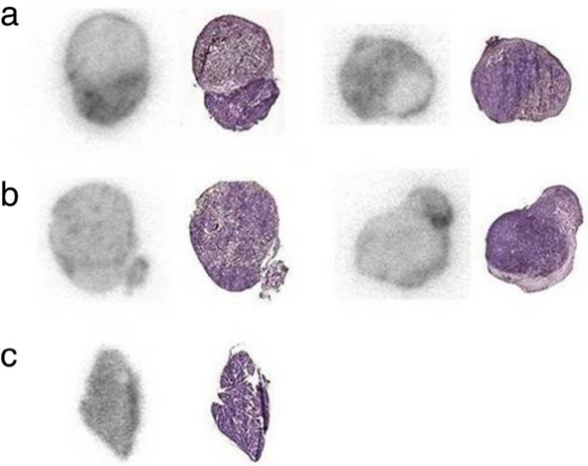
**Autoradiograms and images of H- and Ch2R-stained tumour sections. (a)** A heterogeneous distribution of activity with higher intensities in areas corresponding to a more intense level of staining with H and Ch2R, 48 h (left) and 72 h (right). **(b)** Stained sections and autoradiograms of two tumours resected at 48 h from mice pre-dosed with 0.8 mg of cold 11B6 48 h prior to injection with ^111^In-DTPA-11B6. **(c)** An autoradiogram and stained section of a submandibular gland at 48 h. This normal organ shows less heterogeneity in activity distribution and hematoxylin and Ch2R staining.

**Figure 5:**
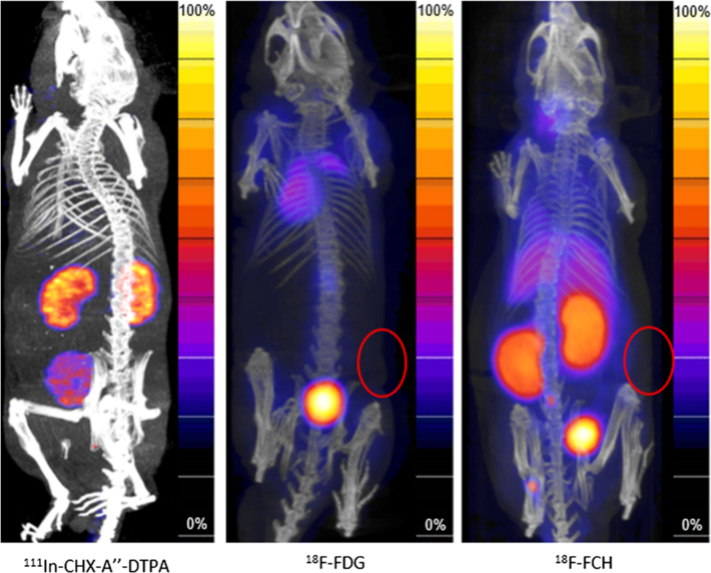
^**111**^ **In-CHX-A″-DTPA SPECT/CT and** ^**18**^ **F-FDG and** ^**18**^ **F-Choline PET/CT images.** SPECT/CT images (left) of non-tumour bearing NMRI nude mice showing localization of ^111^In-CHX-A″-DTPA in the kidneys (0.5 h post-injection). PET/CT images of s.c. LNCaP xenografts 1 h after injection with ^18^ F-FDG and ^18^ F-Choline, respectively (middle and right). Tumours, indicated by red circles, were not well visualized with these radiotracers.

The ROI analysis showed that the ratio of tumour to soft tissue of the contralateral leg was 6.1 ± 1.5 at 48 h (*n* = 4) and 6.8 ± 1.7 at 72 h (*n* = 3). ROI analysis also showed a tumour-to-heart ratio of 1.7 ± 0.7 and a tumour-to-liver ratio of 1.7 ± 0.40 at 48 h and 1.81 ± 0.10 and 1.9 ± 0.04, respectively, at 72 h. This is similar to the tumour-to-tissue ratios derived from the biodistribution data. Interestingly, an obvious heterogeneous distribution of activity could be seen in the SPECT images of the tumours, later confirmed in autoradiograms (Figure 4a). High accumulation of activity was also detected in the salivary glands. For the blocked mice (*n* = 4), the ROI ratio at 48 h of the tumour to the contralateral leg, heart and liver was 3.4 ± 0.82, 1.3 ± 0.41 and 0.87 ± 0.07, respectively. The salivary gland uptake was not associated with detached indium-labelled CHX-A″-DTPA, as confirmed with SPECT/CT (Figure 5).

We also checked whether ^111^In-DTPA-11B6 could be used to detect PCa skeletal lesions. Osseous tumours were established in SCID mice through intra-tibial injections of LNCaP cells in the left hind limb, and SPECT/CT images were performed after i.v. injection of ^111^In-DTPA-11B6. The legs were resected and measured in the NaI(Ti) well counter, using the right non-xenografted legs as a negative control. The data showed an at least twofold uptake of ^111^In-DTPA-11B6 in the tibia compared to the reference leg, some of which might be due to inflammation induced by the surgical procedure. MR imaging confirmed tumour development within the bone (Figure 3b).

Although ^111^In-labelled 11B6 showed a good stability in murine serum, we investigated whether the high uptake seen in the salivary glands could be a result of indium-labelled CHX-A″-DTPA, this was not confirmed (Figure 5, left). PET/CT images of NMRI-nude mice with s.c. LNCaP showed that ^18^F-FDG and ^18^F-Choline were hardly able to visualize the LNCaP xenografts, as compared to ^111^In-DTPA-11B6 (Figure 5, right). ROI analysis showed that the ratio of these xenografts to the soft tissue of the contralateral leg was 1.1 ± 0.38 for ^18^F-FDG (*n* = 4) and 1.09 ± 0.45 for ^18^F-Choline (*n* = 4).

### Autoradiography and staining

Activity accumulation was confirmed to be heterogeneous in tumour sections at both 48 and 72 h. Comparative autoradiograms between the pre-dosed (Figure 4b) and non-pre-dosed mice (Figure 4a) showed that for the former, activity was confined to areas with high hematoxylin and Ch2R staining, i.e. areas of high cell density, whereas the activity in tumours from pre-dosed animals was found in areas with low hematoxylin and Ch2R staining. This difference could be due to an active uptake in the viable regions, blocked in the latter by pre-dosing with cold antibody. The activity distribution was more homogenous in the salivary glands of non-pre-dosed mice, indicating an uptake in all glandular structures in this organ (Figure 4c).

## Discussion

There is a clinical need for improved methods to detect and stage prostate cancer. The imaging of androgen-regulated prostate-specific antigens overexpressed in prostate cancer, such as PSA, would be advantageous for diagnosing and monitoring the disease. In this study, we demonstrated the ability for a novel radiotracer targeting free hK2, a prostate-specific kallikrein homologous to PSA, to specifically target and image hK2 expression in AR- and hK2-positive LNCaP xenografts. Significantly, ^111^In-DTPA-11B6 exhibited strong targeting in both subcutaneous and intra-tibial bone xenografts, with a mean tumour uptake of 19%IA/g at 48 h in subcutaneous tumours (Figure 1a). The elevated uptake of ^111^In-DTPA-11B6 in the salivary glands was an unexpected result of this study; however, several glandular kallikreins are synthesized by the salivary glands in mice [27]. Since we see an uptake in the salivary glands of both NMRI nude and normal non-xenografted NMRI mice, it seems likely that ^111^In-DTPA-11B6 cross-reacts with kallikreins expressed in the salivary glands of these mice.

The tumour accumulation of ^111^In-DTPA-11B6 was hK2-specific, as verified by the low accumulation in the negative control, DU145 xenografts and by competitive binding assays using excess cold non-labelled antibody. Furthermore, tumour accumulation slowly decreased after day 2 but was not as rapid as blood clearance at the same time points. This gave a steadily increasing pattern in tumour-to-blood (T/B) ratios over time, from 1.6 ± 0.41 at 48 h to 2.4 ± 0.62 at 1 week (Table 1). Though the blood clearance of ^111^In-11B6 is comparable to other full-sized IgGs, the high tumour uptake, an increasing T/B ratio and a comparatively low liver uptake, implicates the usefulness of this radiotracer for imaging. Further, most tissues seem to display blood kinetics, except for a faint active uptake by the liver, spleen and lung compared to the high accumulation rate observed in the tumour and salivary gland tissue. Tissues such as the muscle and brain gave high T/Ti ratios of 40 ± 11 and 118 ± 13, respectively, at 1 week, whereas the liver and spleen have lower T/Ti ratios of 4.4 ± 0.28 and 3.7 ± 0.83, respectively. There could be some concerns raised about ^111^In-DTPA-11B6 binding to free hK2 in the blood. However, the blocking with cold non-labelled antibody does not seem to change the amount of the blood activity significantly at 48 h post-injection. Also, in the control group with DU145 xenografts, there was no significant difference in blood activity at 48 h, but a significant difference in tumour uptake was found. One interesting observation from the pre-dosing studies was that the %IA as a function of the tumour size (Figure 2c) differed between the pre-dosed (all pre-dosed/co-injected groups) and non-blocked tumours if these were not too small in tumour size. This explains the large deviation for the pre-dosing at 120 h in Figure 2b. These results also suggest a long tumour retention of the antibody, something also seen in the T/B ratio.

Despite the high sequence homology between PSA and hK2, the 11B6 antibody did not show any cross-reactivity to PSA. This was supported by the fact that co-injection with a high dose of PSA-specific 5A10 antibody did not hinder the uptake of ^111^In-DTPA-11B6 in LNCaP xenografts (Figure 3a). This highlights the possibility of concurrent use. As both *KLK3* and *KLK2* genes are regulated by AR signalling, it is reasonable to theorize that the 11B6 antibody could be used to image AR signalling in prostate cancer in a similar fashion to that reported with 5A10 [18].

In addition, the tracer's specificity was further confirmed by the distinct differences seen between pre-dosed and non-pre-dosed LNCaP xenografts. This difference was also validated by both SPECT and in excised xenografts (Figure 3a, Figure 2). Furthermore, autoradiograms demonstrate that the localization of the labelled antibody to a dense PCa tissue could be changed or blocked by pre-dosing (Figure 4a,b). Although the underlying physiology remains unknown, this could be due to a decrease in an active uptake and/or the blocking of a pool of free hK2 in tumour tissue.

Based on our data, we believe that radiolabelled monoclonal antibodies such as ^111^In-DTPA-11B6 have an immense value for use in the imaging, staging and evaluation of advanced PCa. The favourable T/Ti ratios also suggest that 11B6 could be used as a moiety for delivering high absorbed doses in radioimmunotherapy, whereas ^111^In-radiolabelled 11B6 could potentially be used for patient-individualized dose planning.

## Conclusions

^111^In-DTPA-11B6 is a new radiotracer for SPECT/CT imaging of hK2-expressing prostate cancer. To our knowledge, this is the first study using hK2 as a target for immune imaging. The favourable biokinetics, high tumour accumulation and low normal organ uptake observed with ^111^In-DTPA-11B6 underscore its potential as a novel PCa imaging agent for the detection of metastatic PCa and for monitoring anti-androgen therapy. Because of its immunogenicity, the murine form of 11B6 is not suitable for future clinical trials. Nevertheless, the therapeutic potential of 11B6 in radioimmunotherapy applications is under investigation, as well as a humanised version of 11B6, which is currently in development.

## Additional file

Additional file 1: Figure S1. Surface plasmon resonance. The binding kinetics of 11B6 and DTPA-11B6 analysed by a Biacore 2000 Sensorgram of A. 11B6 to hK2, before conjugation and B. DTPA-11B6 to hK2, after conjugation. ka, kd and KD for 11B6 and DTPA-11B6 are displayed below the graphs.
