# Supplementary material for: Preclinical imaging of kallikrein-related peptidase 2 (hK2) in prostate cancer with a 111In-radiolabelled monoclonal antibody, 11B6
Source: EJNMMI Res. 2014 Sep 19;4:51. doi: 10.1186/s13550-014-0051-5 (PMC4452661; doi:10.1186/s13550-014-0051-5)
Supplement: Additional file 1: Figure S1. — Surface plasmon resonance. The binding kinetics of 11B6 and DTPA-11B6 analysed by a Biacore 2000 Sensorgram of A. 11B6 to hK2, before conjugation and B. DTPA-11B6 to hK2, after conjugation. ka, kd and KD for 11B6 and DTPA-11B6 are displayed below the graphs. [file 13550_2014_51_MOESM1_ESM.pdf]

Preclinical imaging of Kallikrein related peptidase 2, hK2, in prostate cancer with a radiolabelled monoclonal antibody, 11B6

Oskar Vilhelmsson Timmermand[1], David Ulmert[2], Susan Evans-Axelsson[3], Kim Pettersson[4], Anders Bjartell[3], Hans Lilja[2,5,6,7], Sven-Erik Strand[1,8], Thuy A. Tran[8].

[1]Dept. of Medical Radiation Physics, Barngatan 2:1, Lund University, S-211 85, Lund, Sweden.

[2]Department of Surgery (Urology), Memorial Sloan-Kettering Cancer Center, 1275 *York Avenue*, New York, NY 10065, USA.

[3]Dept. of Clin. Sciences, Div. Urological Cancers, Jan Waldenstroms gata 5, Lund University, S-205 02, Malmö, Sweden

[4]Dept. of Biochemistry and Food Chemistry/Biotechnology, Tykistökatu 6A BioCity, University of Turku, 205 20 Turku 52, Turku, Finland.

[5]Department of Laboratory Medicine and Medicine (GU Oncology), Memorial Sloan-Kettering Cancer Center, 1250 York Avenue, New York, NY 10065, USA.

[6]Nuffield Department of Surgical Sciences, University of Oxford, Oxford, United Kingdom.

[7]Department of Laboratory Medicine, Lund University, University Hospital UMAS, Malmö, Sweden

[8]Lund University Bioimaging Center, Lund University, BMC, Klinikgatan 32, S-222 42, Lund, Sweden

SUPPLEMENTARY DATA

A.

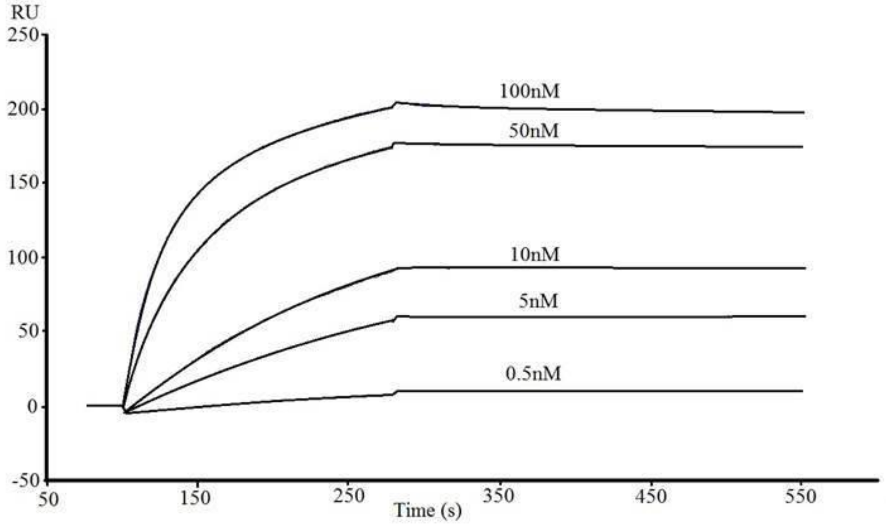

B.

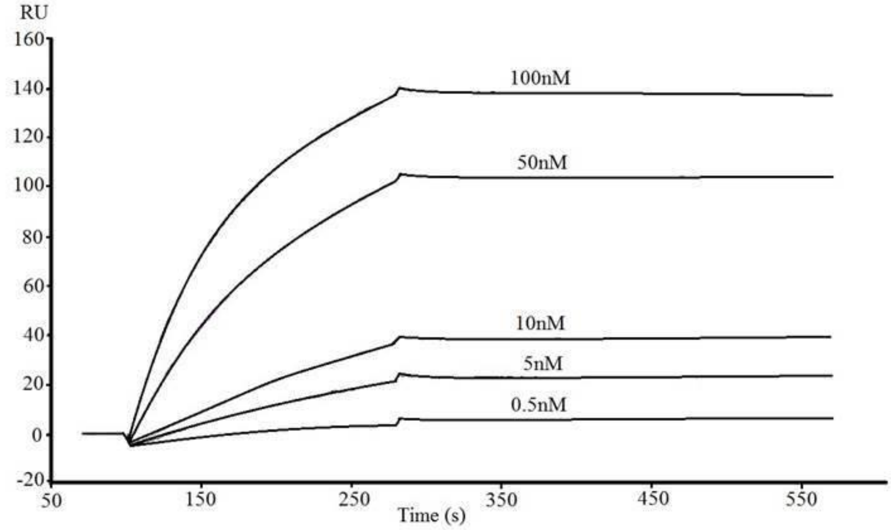

	k_a (1/Ms) (10^5)	k_d (1/s) (10^{-5})	K_D (pM)
A. 11B6	3.77 ± 0.06	1.94±0.08	51.2 ± 21.5
B. DTPA-11B6	1.52 ± 0.11	<1	66.2 ± 4.63

Figure S1. Surface Plasmon Resonance

The binding kinetics of 11B6 and DTPA-11B6 analyzed by a Biacore 2000 Sensorgram of A. 11B6 to hK2, before conjugation and B. DTPA-11B6 to hK2, after conjugation. k_a, k_d and K_D for 11B6 and DTPA-11B6 is displayed below the graphs.
